# Preoperative Prediction of Metastasis for Ovarian Cancer Based on Computed Tomography Radiomics Features and Clinical Factors

**DOI:** 10.3389/fonc.2021.610742

**Published:** 2021-06-10

**Authors:** Yao Ai, Jindi Zhang, Juebin Jin, Ji Zhang, Haiyan Zhu, Xiance Jin

**Affiliations:** ^1^ Department of Radiotherapy Center, The 1st Affiliated Hospital of Wenzhou Medical University, Wenzhou, China; ^2^ Department of Gynecology, The 1st Affiliated Hospital of Wenzhou Medical University, Wenzhou, China; ^3^ Department of Medical Engineering, The 1st Affiliated Hospital of Wenzhou Medical University, Wenzhou, China; ^4^ Department of Gynecology, Shanghai First Maternal and Infant Hospital, Tongji University School of Medicine, Shanghai, China; ^5^ School of Basic Medical Science, Wenzhou Medical University, Wenzhou, China

**Keywords:** ovarian cancer, metastasis, computed tomography, radiomics, CA125

## Abstract

**Background:**

There is urgent need for an accurate preoperative prediction of metastatic status to optimize treatment for patients with ovarian cancer (OC). The feasibility of predicting the metastatic status based on radiomics features from preoperative computed tomography (CT) images alone or combined with clinical factors were investigated.

**Methods:**

A total of 101 OC patients who underwent primary debulking surgery were enrolled. Radiomics features were extracted from the tumor volumes contoured on CT images with LIFEx package. Mann-Whitney *U* tests, least absolute shrinkage selection operator (LASSO), and Ridge Regression were applied to select features and to build prediction models. Univariate and regression analysis were applied to select clinical factors for metastatic prediction. The performance of models generated with radiomics features alone, clinical factors, and combined factors were evaluated and compared.

**Results:**

Nine radiomics features were screened out from 184 extracted features to classify patients with and without metastasis. Age and cancer antigen 125 (CA125) were the two clinical factors that were associated with metastasis. The area under curves (AUCs) for the radiomics signature, clinical factors model, and combined model were 0.82 (95% CI, 0.66-0.98; sensitivity = 0.90, specificity = 0.70), 0.83 (95% CI, 0.67-0.95; sensitivity = 0.71, specificity = 0.8), and 0.86 (95% CI, 0.72-0.99, sensitivity = 0.81, specificity = 0.8), respectively.

**Conclusions:**

Radiomics features alone or radiomics features combined with clinical factors are feasible and accurate enough to predict the metastatic status for OC patients.

## Introduction

Ovarian cancer (OC) is the second most common gynecological cancer and the leading cause of cancer-related death in women with gynecological cancer, with less than 40% patients cured (1). Due to the location of the ovaries, early detection of OC is difficult, with around 70% of patients diagnosed with advanced diseases, which are mainly intra-peritoneal spread and distant metastases (2). Studies indicated that the main pattern of spread of OC is local extension, intra-abdominal, and lymphatic dissemination, with the most common metastatic sites in pleural effusion, liver, abdominal wall, and extra-abdominal lymph nodes (3, 4). Reported five-year survival for these advanced patients is only around 20% to 45% (5).

Early detection of metastasis is critical for the accurate staging of OC and has the potential to improve prognosis, treatment, and survival for OC patients (6). However, no effective screening methods have been established yet, as studies demonstrated that screening methods with cancer antigen 125 (CA125), ultrasound, etc., failed to decrease the mortality from OC (7, 8). Medical imaging is currently frequently applied in the detection and staging for OC. Magnetic resonance imaging (MRI) demonstrates a high accuracy in characterizing primary tumors, peritoneal, and distant staging (9). However, MRI suffers from low sensitivity when the node size is small, and its high cost and longer imaging time also preclude its routine application (10, 11). Positron emission tomography/computed tomography (PET/CT) is also very expensive and has more radiation exposure. The role of PEC/CT in staging for epithelial ovarian cancer (EOC) is still controversial (12). Ultrasound has a lower cost than CT, however, its accuracy is questioned (13). Lymph node metastasis (LNM) is included in the International Federation of Gynecology and Obstetrics (FIGO) staging system for OC, with its well-documented correlation with poor prognosis (14). The detection of lymph node involvement is usually performed with a systematic lymphadenectomy. However, the reported LNM after systematic lymphadenectomy is only around 13.6% to 30.3% for patients with optimally debulked advanced OC (6, 15).

CT is used routinely for OC diagnosis and treatment assessment due to its superior advantages of wide availability, fast scanning time, high cost-efficiency, and good reproducibility (13). CT is also recommended as a standard imaging modality for preoperative staging and follow-up for OC by the European Society of Urogenital Radiology (ESUR) (6). One disadvantage of CT is its low sensitivity, which affects the accuracy of localizing metastatic masses and its ability of characterizing lymph nodes (16). Another limitation of CT is the operator dependence. The imaging acquisition and interpretation are dependent on the radiologist’s experience, which causes great inter- and intra- observe variability in OC staging (17). Therefore, there is an imperative need for an accurate and objective prediction method based on CT to accurately evaluate the metastatic status to help physicians in staging and choosing a more aggressive treatment plan for patients with OC, so as to provide an optimal surgical approach to improve the clinical outcome and the quality of life (18, 19).

With the emergence of radiomics, it is hypothesized that radiomics features derived from images can add relevant information to tumor biology and improve personalized medical decision making (20, 21). The radiomics extracted from MRIs have been used to predict the histologic subtypes and malignancy degree of OC patients, as well as to evaluate the association between the radiomics and survival among EOC (22–24). Studies demonstrated that radiomics features from CT images were associated with heterogeneity across the metastatic lesions of OC, and it is possible to predict the chemotherapy response and disease progression for OC based on CT radiomics (25–27). Accurate preoperative prediction of metastatic status with CT radiomics will help physicians understand the disease better and optimize the decision making for the management of patients with OC. The purpose of this study is to investigate the feasibility of predicting the metastatic status based on radiomics features derived from preoperative CT images alone or combined with clinical factors, so as to help physicians to optimize management for patients with OC.

## Materials and Methods

### Patients

**Figure 1:**
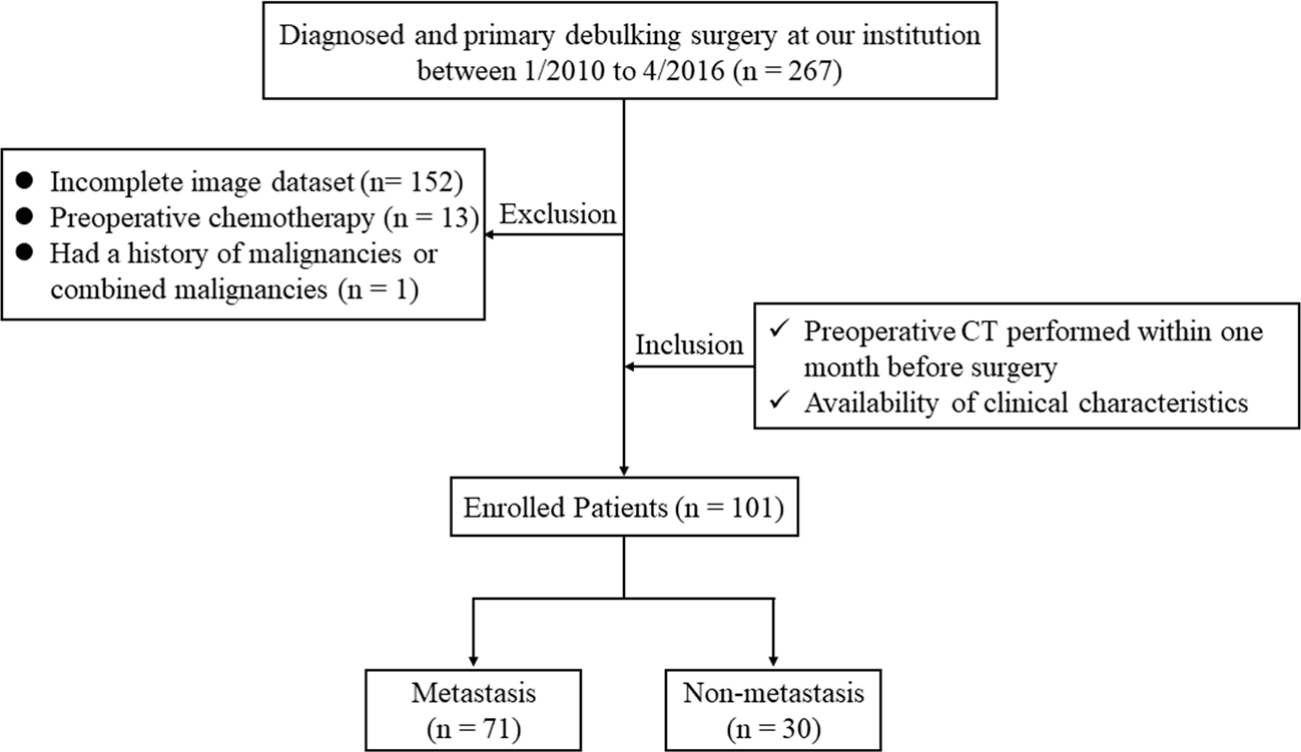
The flowchart of the case identification process.

By searching the electronic medical records, a total of 267 patients who were diagnosed with OC and underwent primary debulking surgery at our hospital between January 2010 and April 2016 were retrospectively reviewed. The inclusion criteria for the study were: patients who underwent routine, unenhanced CT examination within one month before surgery; and available routine clinical evaluation with blood tests. The exclusion criteria were: patients with a lack of digital imaging data (*n* = 152), those treated with preoperative chemotherapy (*n* = 13), and patients with a history of other malignancies or combined malignancies (*n* = 1). Consequently, 101 OC patients with pathologically confirmed metastatic status were enrolled in our study; the main metastatic sites included the uterus, vermix, and intestinal canal. The flowchart of the case identification process was shown in **Figure 1**. Patients treated between January 2010 and May 2014 were assigned to a training cohort (*n* = 70) and patients treated between June 2014 and April 2016 were assigned to a validation cohort (*n* = 31). This retrospective study was approved by the Institutional Review Board of our hospital and conducted in accordance with the Declaration of Helsinki (ECCR no. 2019059), and informed consent was waived by ECCR for the retrospective nature of this study.

### CT Images Acquisition and Tumor Segmentation

CT imaging was acquired at our institute with one of the following CT scanners: BrightSpeed (GE Healthcare, Milwaukee, WI, USA) or Brilliance 16 (Philips Healthcare, Cleveland OH, USA). The scanning parameters were: 120kV, auto tube current, rotation time 0.4 or 0.5s, field of view 300-500 mm, pixel size 512 × 512, slice interval 5 mm, slice thickness 5 mm, and reconstructed section thickness 3 mm. All CT images were retrieved from the picture archiving and communication system (PACS).

**Figure 2:**
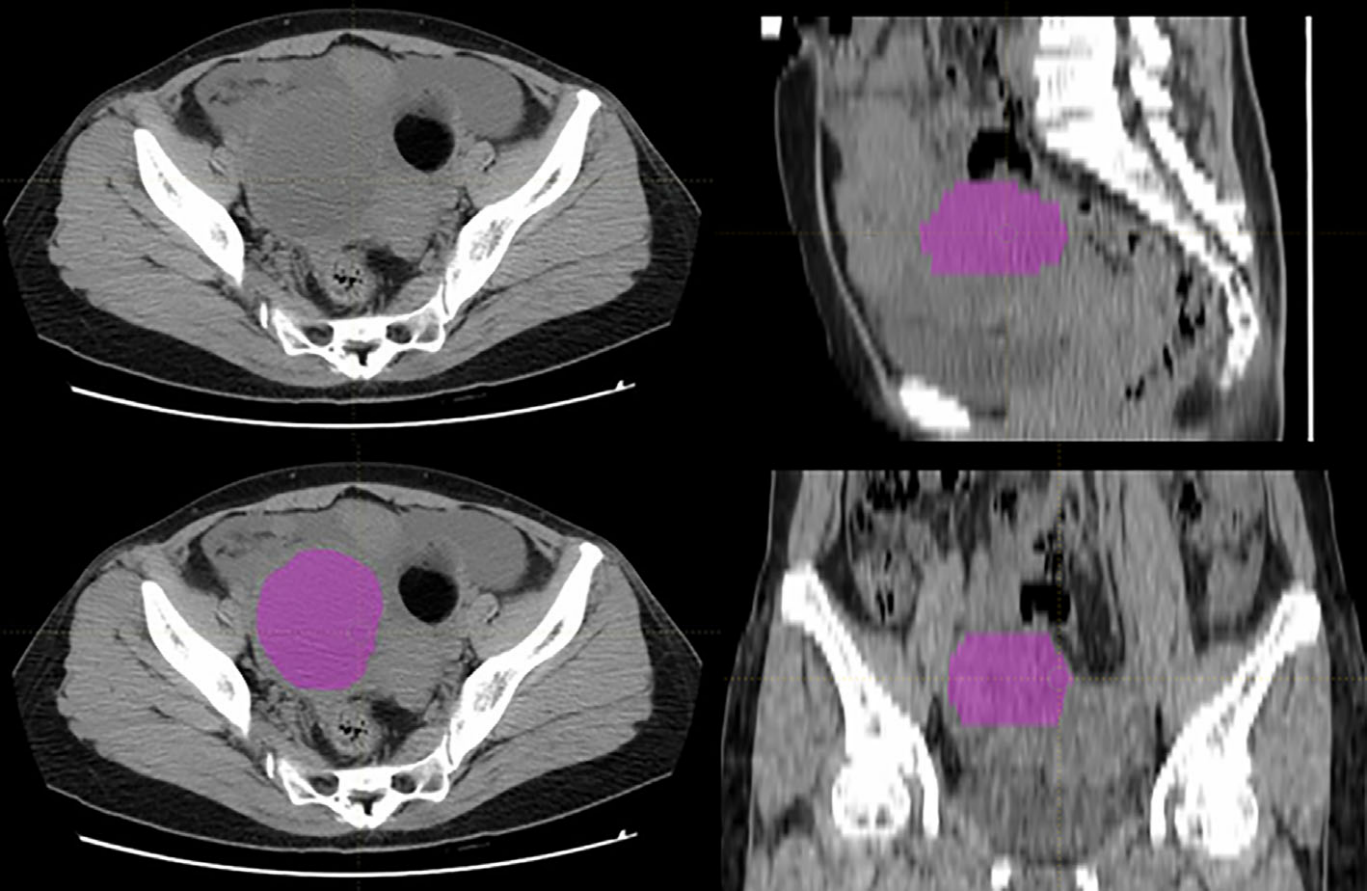
A typical ovarian tumor segmentation on CT images.

The tumor volumes were manually segmented using LIFEx package (http://www.lifexsoft.org) by a radiologist with 7 years of experience in gynecological imaging, and all the segmentations were confirmed by a senior radiologist with over 15 years of experience in gynecological imaging (28). If there were disagreements between the two radiologists, we adopted the idea of the senior radiologist from the point of view of practice experience. The tumor volumes were set as regions of interest (ROIs) for further radiomics feature extraction. A typical ovarian tumor segmentation on CT images was presented in **Figure 2**.

### Radiomics Features Extraction and Model Building

Preprocessing with intensity normalization and spatial resampling was performed for all CT images in LIFEx, which was then used to extract radiomics features. Based on different matrices capturing the spatial intensity distributions at four different scales, a total of 148 radiomic features were extracted from the original images, which contained 23 first-order features derived from histogram, shape, and conventional statistics, and 125 second-order features derived from gray-level co-occurrence matrix (GLCM), neighborhood gray-level different matrix (NGLDM), gray-level run length matrix (GLRLM), and gray-level zone length matrix (GLZLM). The details of the radiomics feature calculation are shown in the **Supplementary Doc. S1**.

A two-step feature selection methodology was employed to reduce the dimension and to select the key features in the training cohort. First, Mann-Whitney *U* tests were applied to select potentially informative features with *P* < 0.05 as statistically significant. Next, by combining the least absolute shrinkage selection operator (LASSO) and the Ridge Regression, “elastic net” was used to identify the optimal features to build the radiomics signature in the training cohort (29). To train and select the optimal prediction model for metastasis of OC, a ten-fold cross-validation was applied to select key features and avoid over-fitting. The final radiomic signature was a linear combination of selected features multiplied by their respective weights. Radiomics score was calculated for each patient. The details were described in the **Supplementary Doc. S2**.

### Clinical Factors and Model Building

Several risk factors had been reported from epidemiological studies for the etiology of OC (30). The clinical factors included in this study were: age, weight, total cholesterol (TCHO) (≤ 5.2 or > 5.2 mmol/L), triglyceride (TG) (≤ 1.7 or > 1.7 mmol/L), high density lipoprotein (HDLC) (≤ 2 or > 2 mmol/L), low density lipoprotein (LDLC) (≤ 3.12 or > 3.12 mmol/L), blood sugar (≤ 6.1 or > 6.1 mmol/L), CA125 (≤ 35 or > 35 U/ml), and carcinoembryonic antigen (CEA) (≤ 5 or > 5 ng/ml). The threshold values for TCHO, TG, HDLC, LDLC, blood sugar, CA125, and CEA levels were decided based on the normal ranges used at our institute.

The univariate analysis was applied to select the clinical factors for metastatic prediction. Categorical variables were compared by using the chi-square test or Fisher exact test. Continues variables were compared by using the Student *t* test or Mann-Whitney *U* test. Variables with a *P* value < 0.05 were used to build the clinical model with logistic regression. The combined model integrating the radiomics features and clinical factors was built using logistic regression.

### Model Evaluation and Statistical Analysis

To assess the predictive ability of the radiomics signature, clinical factor model, and combined model, receiver operating characteristics (ROC) curves and predicted value were generated in the training cohort and validation cohort, respectively. The area under the curves (AUCs) was used to evaluate the accuracy of these models in predicting the metastasis in both the training cohort and validation cohort, respectively.

Statistical analysis was performed using R analysis platform (version 3.6.0) and OriginPro2016. LASSO logistic regression model building was done using the “glmnet” package. Categorical variables were compared by using the chi-square test. Continuous variables were compared by using the Mann-Whitney *U* test. For all tests, *P*< 0.05 was thought statically significant. All statistical analyses of this study were confirmed by two statisticians who are experienced in statistical analysis of medical data.

## Results

### Patients’ Characteristics

The details of demographic statistics of patients enrolled in this study were presented in **Table 1**. The median age of the enrolled 101 patients was 54.23 years (range from 15-79), with 71 (70.3%) patients having confirmed metastasis at surgery. More than half of the patients (56.4%) were found with stage III. EOC was found in 86 (85.1%) patients, and 12 (11.9%) presented with vascular invasion. **Table 2** presents the main metastatic sites and the number of metastases of the enrolled patients in this study. There was a total of 100 metastases for the enrolled 71 metastatic patients, in which intestinal canal and omentum majus were the two sites with most metastases.

**Table 1:** Characteristics of patients.

Characteristics	Metastasis (-) (*n* = 30)	Metastasis (+) (*n* = 71)	*P*
Age (years), mean (range)	46.9 (15-76)	57.3 (26-79)	<0.001
FIGO stage			<0.001
I	29	0	
II	1	13	
III	0	57	
IV	0	1	
Histological type			<0.001
Epithelial	19	67	
Non-epithelial	11	4	
Vascular invasion			0.016
Yes	0	12	
No	30	59	

Categorical variables were compared by using the chi-square test or Fisher exact test. Continues variables were compared by using the Student t test or Mann-Whitney U test.

FIGO, International Federation of Gynecology and Obstetrics.

**Table 2:** The main metastatic sites and number of metastases of enrolled patients with ovarian cancer.

The main site of metastasis	Uterus	Vermix	Intestinal canal	Omentum majus	Lymph nodes
Numbers of metastases	18	9	30	30	13
Percentage	25.4%	12.7%	42.3%	42.3%	18.3%

### Radiomics Features Selection and Signature Building

There were 43 out of 148 extracted radiomics features selected according to the Mann-Whitney U test with a *P* < 0.05. Nine radiomics features were further screened out from the 43 features to classify patients with metastasis based on the results of elastic net after the parameter tuning during regression analysis. These features included one GLRLM feature, six GLZLM features, and two NGLDM features.

The radiomics signature was established by the linear combination of the selected features using the LASSO regression, and the radiomics score for each patient was calculated in the training cohort and validation cohort, respectively. The detail of the radiomics score calculation formula was shown in the **Supplementary Doc. S3**.

### Clinical Factors for Metastatic Prediction

The univariate analysis of preoperative clinical factors associated with metastasis in the training and validation cohorts was presented in **Table 3**. The results indicated that age and CA125 levels were two risk factors of metastasis for patients with OC. No other risk factor was shown in both the training and validation cohorts.

**Table 3:** Univariate analysis of preoperative clinical factors associated with metastasis.

Characteristics	Training cohort		*P*	Validation cohort		*P*
	Metastasis (–) (*n* = 20)	Metastasis (+) (*n* = 50)		Metastasis (-) (*n* = 10)	Metastasis (+) (*n* = 21)	
Age (years),			0.012*			0.018*
Mean (range)	48.4 (23-76)	57.9 (29-79)		43.8 (15-61)	56.1 (26-70)	
Weight (kg)			0.92			0.06
Mean (range)	57.0 (47-75)	56.9 (42-76)		60.1 (51-66)	57.2 (43-81)	
TCHO (mmol/L)			0.59			0.25
≤5.2	11	31		4	13	
>5.2	9	19		6	8	
TG (mmol/L)			0.73			0.70
≤1.7	14	37		7	16	
>1.7	6	13		3	5	
HDLC (mmol/L)			0.11			0.74
≤2	19	50		9	18	
>2	1	0		1	3	
LDLC (mmol/L)			0.70			0.05
≤3.12	13	30		4	16	
>3.12	7	20		6	5	
Blood sugar (mmol/L)			0.88			0.21
≤6.1	10	26		8	12	
>6.1	10	24		2	9	
CA125 (U/ml)			< 0.001*			0.001*
≤35	7	2		6	1	
>35	13	48		4	20	
CEA (ng/ml)			0.38			0.95
≤5	15	32		8	17	
>5	5	18		2	4	

*P value < 0.05; Categorical variables were compared by using the chi-square test. Continues variables were compared by using the Student t test or Mann-Whitney U test;

TCHO, total cholesterol; TG, triglyceride; HDLC, high density lipoprotein; LDLC, low density lipoprotein; CA125, carcinoma antigen 125; CEA, carcinoembryonic antigen.

### Models Performance

**Figure 3:**
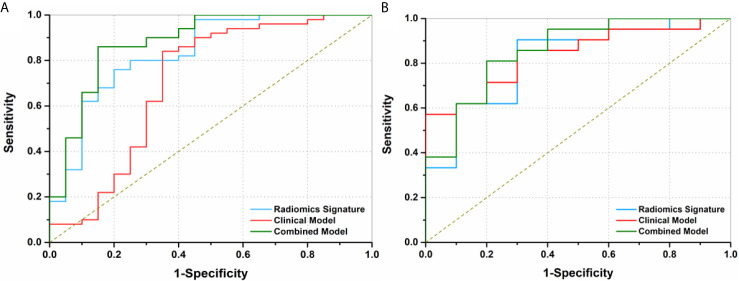
Receiver operating characteristics (ROC) curves of three models to discriminate metastasis in preoperative ovarian cancer patients. **(A)** The area under curves (AUCs) of radiomics signature, clinical factor model, and combined model were 0.84 (95% CI, 0.74-0.93), 0.70 (95% CI, 0.58-0.83) and 0.88 (95% CI, 0.80-0.96) in the training cohort, respectively**. (B)** The AUCs of the radiomics signature, clinical model, and combined model were 0.82 (95% CI, 0.66-0.98), 0.83 (95% CI, 0.67-1.00), and 0.86 (95% CI, 0.72-0.99) in the validation cohort, respectively.

**Figure 4:**
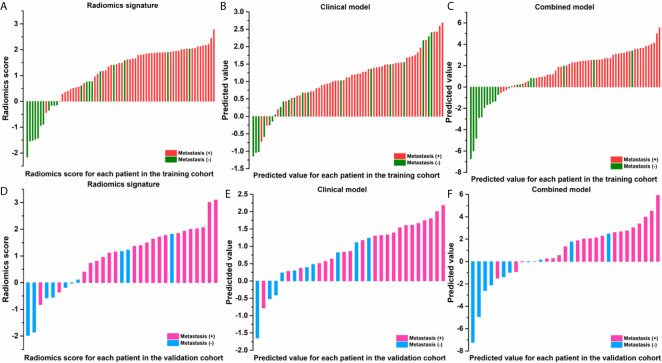
Radiomic score for each patient in the training and validation cohorts of radiomic signature **(A, D)**; of clinical model **(B, E)**; of combined model **(C, F)**.

The ROCs and AUCs of the radiomics signature, clinical model, and combined model in the training and validation cohorts were shown in **Figure 3** and **Table 4**. The AUCs of the radiomics signature were 0.84 [95% confidence interval (CI), 0.74-0.93; sensitivity = 0.76, specificity = 0.80] with a cutoff of 1.11 in the training cohort and 0.82 (95% CI, 0.66-0.98; sensitivity = 0.90, specificity = 0.70) in the validation cohort, respectively. The AUCs of the clinical model were 0.70 (95% CI, 0.58-0.83; sensitivity = 0.84, specificity = 0.65) with a cutoff of 0.70 in the training and 0.83 (95% CI, 0.67-0.95; sensitivity = 0.71, specificity = 0.8) in the validation cohorts, respectively. The combined model showed a valuable predictive performance with AUCs of 0.88 (95% CI, 0.80-0.96, sensitivity = 0.86, specificity = 0.85) with a cutoff of 0.82 in the training cohort and 0.86 (95% CI, 0.72-0.99, sensitivity = 0.81, specificity = 0.8) in the validation cohort, respectively. The results of radiomics score for each OC patient in the training and validation cohorts were shown in **Figure 4**. The radiomics scores and predicted values in each model for patients with metastatic status were higher than those for patients with negative metastatic status both in the training cohort and validation cohort.

**Table 4:** Predictive performance of the radiomics signature and the two prediction models for the discrimination of metastasis in ovarian cancer.

Models	Training cohort			Validation cohort		
	SEN	SPE	AUC (95% CI)	SEN	SPE	AUC (95% CI)
Radiomics model	0.76	0.80	0.84 (0.74-0.93)	0.90	0.70	0.82 (0.66-0.98)
Clinical model	0.84	0.65	0.70 (0.58-0.83)	0.71	0.80	0.83 (0.67-0.95)
Combined model	0.86	0.85	0.88 (0.80-0.96)	0.81	0.80	0.86 (0.72-0.99)

SEN, sensitivity; SPE, specificity; AUC, are under curve; 95% CI, 95% confidence interval.

## Discussion

In this study, the feasibility of CT-based radiomics in the prediction of metastasis of OC had been investigated. Reasonable prediction accuracy with an AUC of 0.82, 0.83, and 0.86 were achieved for radiomics feature signature, clinical factor model, and combined model, respectively. The radiomics scores and predicted values in each model for patients with metastasis status were higher than those for patients with negative metastasis status, as shown in **Figure 4**. For patients with a higher radiomics scores or predicted values compared with the cutoff values implies a high risk of metastasis. Therefore, the physicians can compare the radiomics scores or predicted values with the cutoff values to assess metastasis status for OC patients preoperatively.

There were 71 out of the enrolled 101 patients with metastases. This is consistent with reports of more than 70 percent of OC patients diagnosed with advanced diseases. Ovarian neoplasms have several histopathologic subtypes, where epithelial ovarian counts for nearly 90% and is the leading cause of cancer-related death for patients with gynecologic cancer (31, 32). In this study, the epithelial subtype comprises 85.1% of the enrolled patients. There were 13 patients (12.9%) with stage II classified into metastases. This is also consistent with the reported 6.1%-29.6% of patients with clinical stage I-II OC and lymph node involvement (33).

Although the sensitivity of CA125 in the detection and monitoring of OC has been questioned (34, 35), it is still a valuable prognostic factor for subtype, recurrence, stage, disease progression, and survival (36). The histological type, grade, and CA125 level of patients with OC at diagnosis are important indicators for systematic lymphadenectomy (37). Recently, an equation consisting of CA125 and age was evaluated for the prediction of metastasis for OC in intention of improving cancer care in low/middle income countries where availability of cross-section imaging was limited, and a sensitivity of 0.82 and specificity of 0.80 were reported (38). Similarly, in this study, we observed an AUC of 0.83 for models with CA125 and age in the prediction of metastasis with a sensitivity and specificity of 0.71 and 0.80, respectively.

The guidelines of ESUR and the American College of Radiology use CT as a standard imaging modality for staging and follow-up for patients with OC (39). With the high-throughput extraction of quantitative features from CT images with radiomics technique, CT images had been applied to evaluate the clinical outcomes through radiomics features from multiple metastatic lesions for patients with high-grade serous OC (25), as well as to predict the disease progression within 12 months for OC patients with residual tumor at surgery (27). However, few studies use CT radiomics for the prediction of metastatic status of OC, to the best of our knowledge.

An AUC of 0.82 was achieved for the CT radiomics feature model in the prediction of metastasis of OC in this study. The AUC was further improved to 0.86 when the radiomics features were combined with clinical factors of CA125 and age. Both results were better than that found in a previous study, in which an AUC of 0.789 was reported when combining the diameter of cardiophrenic lymph nodes (CPLN), the short and long axes, and clinical factors of age and CA125 to predict the CPLN metastasis for patients with advanced EOC (40). The advantage of using radiomics features from ovarian tumors directly to predict the metastatic status is that the insensitivity of images in the classification of lymph nodes with small diameters can be overcome.

One main limitation of this study is that this is a retrospective study carried out in a single center. The number of patients enrolled was relatively small. A multicenter study with a larger population of patients is needed to further validate the findings in this study. Radiomics features extracted from other image modalities, such as MRI and PET/CT, are also valuable to further validate this study.

## Conclusions

The demonstrated feasibility and accuracy of metastatic status prediction methods, which combined radiomics features from preoperative CT with CA125 and age, provide a noninvasive method to help physicians to accurately evaluate the metastatic status and optimize the treatment for patients with OC.

## Data Availability Statement

The original contributions presented in the study are included in the article/**Supplementary Material**. Further inquiries can be directed to the corresponding authors.

## Ethics Statement

This study is conformed to the guidelines of the Declaration of Helsinki and was approved by the Institutional Review Board of The First Affiliated Hospital of Wenzhou Medical University (ECCR no. 2019059).

## Author Contributions

Conception and design: HZ and XJ. Administrative support: YA and JDZ. Provision of study materials or patients: YA and JDZ. Collection and assembly of data: YA and JDZ. Data analysis and interpretation: JZ and JJ. Manuscript writing: YA, JDZ, HZ, and XJ. Final approval of manuscript: YA, HZ, and XJ. All authors contributed to the article and approved the submitted version.

## Funding 

This work was supported by Wenzhou Municipal Science and Technology Bureau (No. 2018ZY016 and Y20190183), Zhejiang Engineering Research Center of Intelligent Medicine(2016E10011), and the National Natural Science Foundation of China under Grant (No. 11675122).

## Conflict of Interest

The authors declare that the research was conducted in the absence of any commercial or financial relationships that could be construed as a potential conflict of interest.

## Abbreviations

OC, ovarian cancer; CT, computed tomography; LASSO, least absolute shrinkage selection operator; CA125, age and cancer antigen 125; AUCs, are under curves; MRI, magnetic resonance imaging; PET/CT, positron emission tomography/computed tomography; EOC, epithelial ovarian cancer; LNM, Lymph node metastasis; FIGO, International Federation of Gynecology and Obstetrics; ESUR, European Society of Urogenital Radiology; ROIs, regions of interest; GLCM, gray-level co-occurrence matrix; NGLDM, neighborhood gray-level different matrix; GLRLM, gray-level run length matrix; GLZLM, gray-level zone length matrix; TCHO, total cholesterol; TG, triglyceride; HDLC, high density lipoprotein; LDLC, low density lipoprotein; CEA, carcinoembryonic antigen; SEN, sensitivity; SPE, specificity; AUC, are under curve; 95% CI, 95% confidence interval.
