# Supplementary material for: Preoperative Prediction of Metastasis for Ovarian Cancer Based on Computed Tomography Radiomics Features and Clinical Factors
Source: Front Oncol. 2021 Jun 10;11:610742. doi: 10.3389/fonc.2021.610742 (PMC8222738; doi:10.3389/fonc.2021.610742)
Supplement: Supplementary file 1 [file DataSheet_1.docx]

**Supplementary Materials**

**Doc. S1: Radiomic feature extraction**

**Doc. S2: Radiomic signature building**

**Doc. S3: Radiomic signature calculation formula**

**Doc. S1: Radiomic feature extraction**

1. **First Order Features**
   1. **Indices from shape**

- 1. **Indices from histogram**

- 1. **Conventional Indices**

$$CONV_{mean}=\frac{1}{N}\sum_{i} Value_{i}$$

$$CONV\_TLG(ml)=V\cdot\frac{1}{N}\sum_{i} SUV_{i}$$

1. **Texture Features**
   1. **GLCM definition**

The grey level co-occurrence matrix (GLCM) takes into account the arrangements of pairs of voxels to calculate textural indices.

- 1. **NGLDM definition**

The neighborhood grey-level different matrix (NGLDM) corresponds to the difference of grey-levels between one voxel and its 26 neighbours in 3 dimensions.

- 1. **GLRLM definition**

The grey-level run length matrix (GLRLM) gives the size of homogeneous runs for each grey-level.

- 1. **GLZLM definition**

The grey-level zone length matrix (GLRLM) provides information on the size of homogeneous zones for each grey-level in 3 dimensions.

$$GLZLM\_SZE=\frac{1}{H}\sum_{i} \sum_{j} \frac{GLZLM(i,j)}{j^{2}}$$

$$GLZLM\_LZE=\frac{1}{H}\sum_{i} \sum_{j} GLZLM(i,j)\cdot j^{2}$$

$$GLZLM\_LGZE=\frac{1}{H}\sum_{i} \sum_{j} \frac{GLZLM(i,j)}{i^{2}}$$

$$GLZLM\_HGZE=\frac{1}{H}\sum_{i} \sum_{j} GLZLM(i,j)\cdot i^{2}$$

$$GLZLM\_SZLGE=\frac{1}{H}\sum_{i} \sum_{j} \frac{GLZLM(i,j)}{i^{2}\cdot j^{2}}$$

$$GLZLM\_SZHGE=\frac{1}{H}\sum_{i} \sum_{j} \frac{GLZLM(i,j)\cdot i^{2}}{j^{2}}$$

$$GLZLM\_LZLGE=\frac{1}{H}\sum_{i} \sum_{j} \frac{GLZLM(i,j)\cdot j^{2}}{i^{2}}$$

$$GLZLM\_LZHGE=\frac{1}{H}\sum_{i} \sum_{j} GLZLM(i,j)\cdot i^{2}\cdot j^{2}$$

$$GLZLM\_GLNUz=\frac{1}{H}\sum_{i} (\sum_{j} GLZLM(i,j))^{2}$$

$$GLZLM\_ZLNU=\frac{1}{H}\sum_{j} (\sum_{i} GLZLM(i,j))^{2}$$

$$GLZLM\_ZP=\frac{H}{\sum_{i} \sum_{j} (j\cdot GLZLM(i,j))}$$

**Doc. S2: Radiomic signature building**


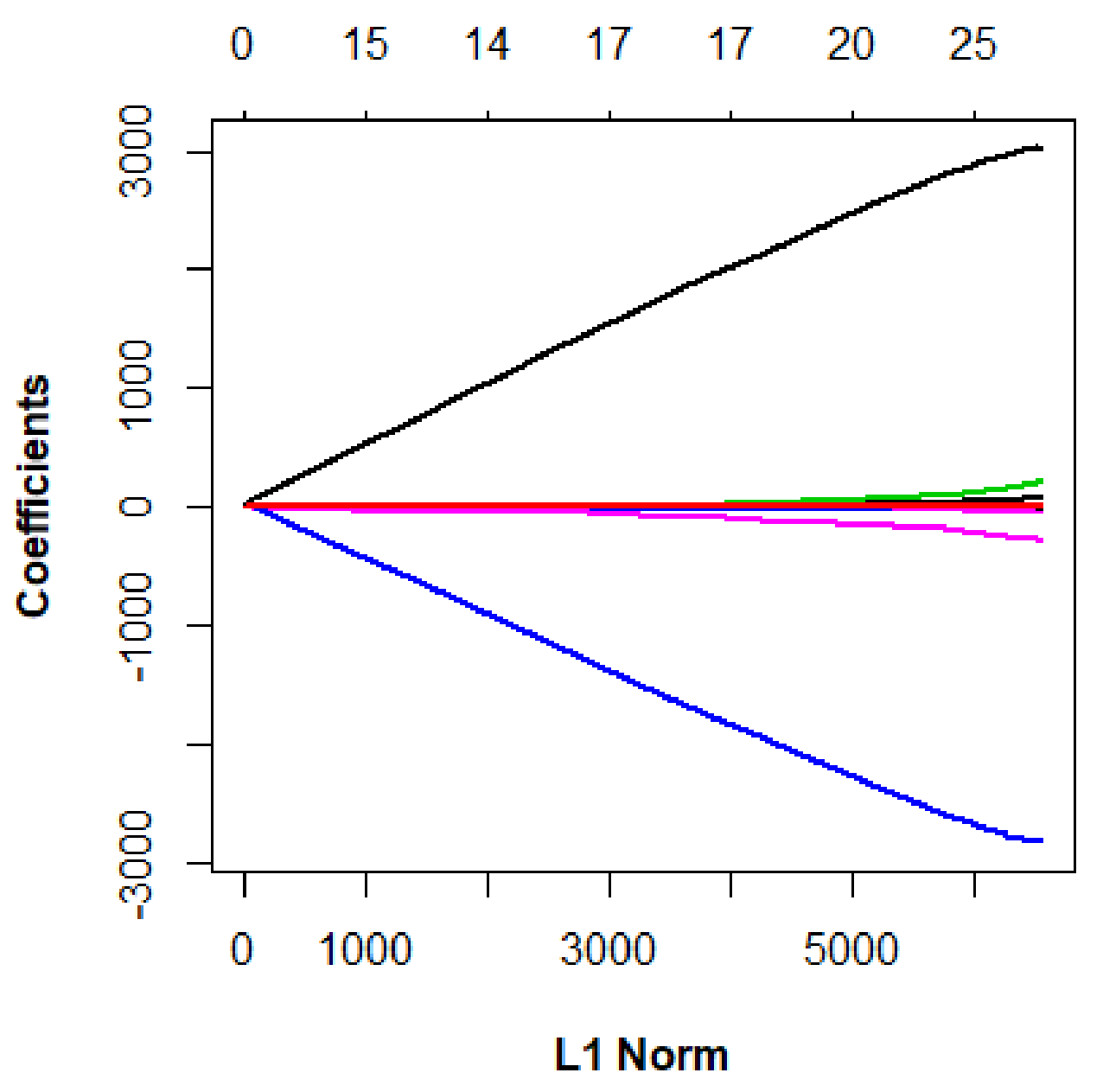

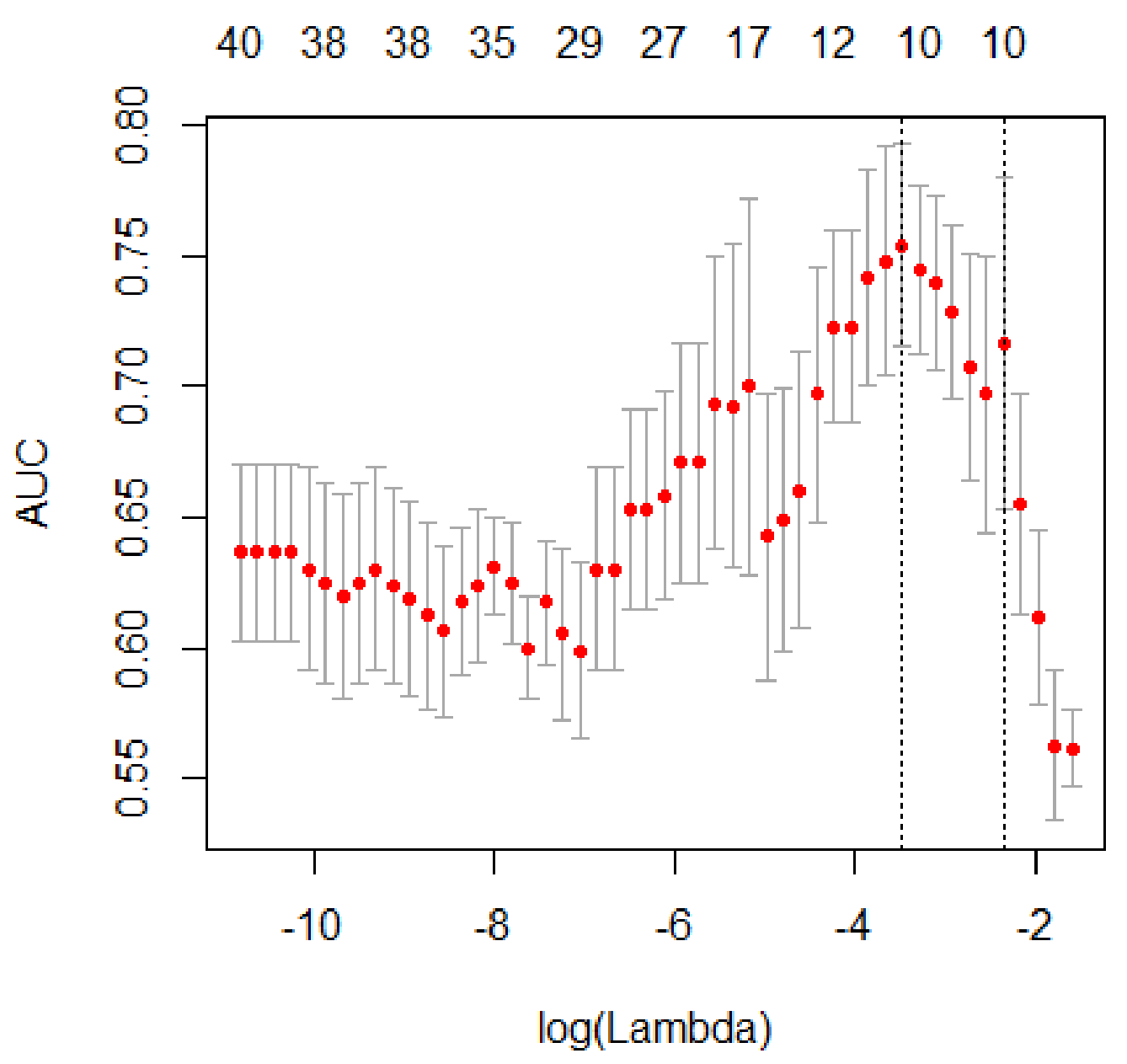


To select key features from high dimensional feature space and avoid over-fitting, ten-fold cross validation was used in the parameters tuning of the elastic net. For tuning coefficient λ, the maximum area under receiver operating characteristic curve (AUC) and criterion of minimum standard deviation were followed. Finally, nine features were chosen to build the radiomics signature.

**Doc. S3: Radiomic signature calculation formula**

$$\begin{matrix} Radiomcis score=1.963+(-3.161)*GLRLM\_LRE \\ + 291.089\cdot*NGLDM\_Coarseness \\ +(-0.0186)*NGLDM\_Busyness \\ +0.0000195 *GLZLM\_ZLNU\_1 \\ +3.551*GLZLM\_ZP\_1 \\ +(-0.0000906)*GLZLM\_GLNU\_2 \\ +0.0000427*GLZLM\_ZLNU\_2 \\ +(-0.0000201)*GLZLM\_GLNU\_3 \\ +(-0.0000737)*GLZLM\_ZLNU\_3 \end{matrix}$$
